# Persistence of Psychological Distress in Surgical Patients with Interest in Psychotherapy: Results of a 6-Month Follow-Up

**DOI:** 10.1371/journal.pone.0051167

**Published:** 2012-12-05

**Authors:** Léonie F. Kerper, Claudia D. Spies, Maria Lößner, Anna-Lena Salz, Sascha Tafelski, Felix Balzer, Edith Weiß-Gerlach, Tim Neumann, Alexandra Lau, Heide Glaesmer, Elmar Brähler, Henning Krampe

**Affiliations:** 1 Department of Anesthesiology and Intensive Care Medicine, Campus Charité Mitte and Campus Virchow-Klinikum, Charité – University Medicine Berlin, Berlin, Germany; 2 Department of Medical Psychology and Medical Sociology, University of Leipzig, Leipzig, Germany; Federal University of Rio de Janeiro, Brazil

## Abstract

**Objectives:**

This prospective observational study investigated whether self-reported psychological distress and alcohol use problems of surgical patients change between preoperative baseline assessment and postoperative 6-month follow-up examination. Patients with preoperative interest in psychotherapy were compared with patients without interest in psychotherapy.

**Methods:**

A total of 1,157 consecutive patients from various surgical fields completed a set of psychiatric questionnaires preoperatively and at 6 months postoperatively, including Patient Health Questionnaire-4 (PHQ-4), Brief Symptom Inventory (BSI), Center for Epidemiologic Studies Depression Scale (CES-D), World Health Organization 5-item Well-Being Index (WHO-5), and Alcohol Use Disorder Identification Test (AUDIT). Additionally, patients were asked for their interest in psychotherapy. Repeated measure ANCOVA was used for primary data analysis.

**Results:**

16.7% of the patients were interested in psychotherapy. Compared to uninterested patients, they showed consistently higher distress at both baseline and month 6 regarding all of the assessed psychological measures (p’s between <0.001 and 0.003). At 6-month follow-up, neither substantial changes over time nor large time x group interactions were found. Results of ANCOVA’s controlling for demographic variables were confirmed by analyses of frequencies of clinically significant distress.

**Conclusion:**

In surgical patients with interest in psychotherapy, there is a remarkable persistence of elevated self-reported general psychological distress, depression, anxiety, and alcohol use disorder symptoms over 6 months. This suggests high and chronic psychiatric comorbidity and a clear need for psychotherapeutic and psychiatric treatment rather than transient worries posed by facing surgery.

## Introduction

Few studies have investigated psychological distress in surgical patients. With the exception of two earlier large-scale investigations [1], [2] research is mostly based on small samples, distinct surgical fields and specific psychological factors. Taken together, there is some evidence that psychological distress is high in surgical patients during the pre- and perioperative period regarding depression, e.g. [3]–[7], anxiety, e.g. [1], and alcohol use disorders, e.g. [8], [9]. However, it is not clear to which extent elevated self-reported symptoms of preoperative psychological distress reflect either clinically significant psychiatric symptoms or transient worries posed by facing surgery. O’Hara et al (1989) found in a large sample study that the rate of patients with clinically significant psychological distress was even higher 3 months after surgery than at the day before surgery [1]. Recent investigations of smaller samples and with follow-up times ranging from 3 days to 3 to 5 years show a differentiated picture: Some studies confirmed the increase of psychological distress [10], [11], others found no significant change [12]–[14], a significant decrease [15]–[18], patterns of no significant change and decrease [19], [20], or patterns of both increase and decrease [21], [22]. In a recent study, we examined N = 4,568 surgical patients in the preoperative anesthesiological assessment clinic and found a rate of clinically significant preoperative psychological distress of up to 38% [23]. Independently of surgical field or physical health, interest in psychotherapy was significantly associated with the intensity of self-reported symptoms of general psychological distress, depression, anxiety and substance use disorders. However, only a prospective longitudinal investigation will provide data to clarify whether elevated symptoms remain stable over time or decrease after patients have overcome the hospital stay.

This study investigated whether self-reported psychological distress and alcohol use problems of surgical patients change between preoperative baseline assessment and postoperative 6-month follow-up examination. Patients with preoperative interest in psychotherapy were compared with patients without interest in psychotherapy. In order to control for types of questionnaire, a set of 12 standardized psychological scales and subscales, respectively, was applied.

## Materials and Methods

### Design and Setting

This prospective observational study was carried out from April 2009 to December 2010 as part of a feasibility study investigating Bridging Intervention in Anesthesiology (BRIA; approved by the local Ethics Committee [EA1/23/2004, Amendment April 2009]), which is currently followed by a randomized controlled trial. Baseline assessment of the feasibility study was performed in the preoperative assessment clinics of the Charité – University Medicine Berlin, and postoperative 6-month follow-up was carried out as a postal questionnaire investigation.

BRIA has been designed as a psychotherapeutic stepped care approach to reach patients from different surgical fields. The program consists of two major therapeutic elements: 1) A computer assisted self-assessment of social, lifestyle and psychological factors including a comprehensive battery of psychiatric screening instruments, items concerning interest in psychotherapy, as well as computerized tailored brief written advice, and; 2) Psychotherapeutic contacts with the objective either to motivate patients with psychiatric disorders and support them in participating in subsequent outpatient psycho- and addiction therapy, or to improve the patients’ psychological symptoms and well-being so that a subsequent psychosocial treatment is not necessary. As previously reported [23], the primary objective of BRIA is to bridge the gap between inpatient surgical treatment and outpatient psychosocial health care including psychotherapy, psychiatry, and addiction treatment.

During the pilot phase of BRIA (April to December 2009), the treatment program was introduced in the preoperative assessment clinics and the computer-assisted self-assessment took place approximately two to three days per week between 9.00 am and 5.00 pm. In the following implementation phase (January to June 2010) BRIA was integrated in the routine care of the hospital so that the computer assisted self-assessment was performed from Monday to Friday between 9.00 am and 5.00 pm in order to cover the complete opening hours of the assessment clinics. Surgical patients examined by an anesthesiologist in the preoperative assessment clinics were assessed for inclusion and exclusion criteria and, in case of eligibility, asked for participation in the study. Inclusion and exclusion criteria were defined as follows. Inclusion criteria: Patient in preoperative anesthesiological assessment clinic, sufficient knowledge of German language, age ≥18 years, written informed consent. Exclusion criteria: Surgery with an emergency or urgent indication; inability to attend the preoperative assessment clinic (bedside visit); members of the hospital staff; relatives of the study team; study participation in another clinical trial; homelessness; admitted in police custody; unwilling to use or incapable of using a computer. Upon receipt of written informed consent, eligible patients completed the computer-assisted self-assessment which took approximately 25 minutes per patient. During the pilot phase (April to December 2009), 1,500 patients were enrolled, and during the implementation phase (January to June 2010) 3,068 patients. Detailed information on the inclusion process is available for the implementation phase: A total of 7,178 patients were assessed for eligibility, with 4,110 not being eligible according to the inclusion/exclusion criteria and 953 refusing to participate (details in [23]). Because the primary objective of the feasibility study was to include as many patients as possible in the baseline assessment, inclusion and exclusion criteria were set rather general. As a consequence, it was no exclusion criterion when patients did not want to participate in the 6-month follow-up and they were only asked to indicate whether they would agree to be contacted by the researchers after 6 months. In total, 4,568 patients participated in both pilot phase and implementation phase of BRIA. 1,838 patients did not show any interest in the 6-month follow-up. Of the remaining 2,730 patients, 1,533 did not send back the follow-up questionnaire and 12 were not reachable because the address was unknown. Of 26 patients information was received that they had died during hospital stay or follow-up period, and 2 patients sent back the questionnaire but did not fill in, resulting in 1,157 patients who participated in both baseline and 6-month follow-up.

### Measurements

A set of standardized screening questionnaires with sound psychometric properties covered the domains of general psychiatric distress, depression, well-being, generalized anxiety and alcohol use disorders: Patient Health Questionnaire-4 (PHQ-4 [24], [25]), Brief Symptom Inventory (BSI [26]), Center for Epidemiologic Studies Depression Scale (CES-D [27]), World Health Organization 5-item Well-Being Index (WHO-5 [28]), and Alcohol Use Disorder Identification Test (AUDIT [29], [30]). Details of the questionnaires are described in Table 1. Additional single-item questions dealt amongst others with demographic information, subjective health status (visual analogue scale of the EuroQol 5 Dimensions, EQ-5D [31]), as well as interest in psycho- and/or addiction therapy sessions of BRIA (“Would you like to have psychotherapy sessions/addiction counselling during your hospital stay?”).

**Table 1:** Standardized self-report questionnaires that were assessed at baseline (T1) and 6-month follow-up (T2).

Name	Description
Patient Health Questionnaire-4: PHQ-4 [24]	
	Ultra-brief screening tool: Subscales for depression (PHQ-2), anxiety (GAD-2), 1 single-item for impairment rating.
	Domains: Depression, anxiety; time frame: Past 14 days.
	5 items, 4-point Likert scale from 0 to 3; for PHQ-2 and GAD-2 each 2 items, ranges from 0 to 6.
	Cut off score: PHQ-2 sum score: ≥3; GAD-2 sum score: ≥3 [24], [25].
Center for Epidemiologic Studies Depression Scale:CES-D [27]	
	Short version of the CES-D: Frequency of depressive symptoms.
	Domain: Depression; time frame: Past 7 days.
	15 items, 4-point Likert scale from 0 to 3; total sum score from 0 to 45.
	Cut off score: CES-D sum score: ≥18 [27].
Brief Symptom Inventory: BSI [26]	
	Short version of the Symptom Checklist 90-R (SCL-90-R): Severity of psychiatric symptoms.
	Domains: General and specific psychological distress; time frame: Past 7 days.
	53 items, 5-point Likert scale from 0 to 4; total mean score from 0 to 4. Applied scores in this study: Global severity index (GSI), subscales depression, anxiety, interpersonal sensitivity, phobic anxiety.
	Cut off score for GSI, depression, anxiety, interpersonal sensitivity, phobic anxiety: T scores: ≥0.63 [26].
World Health Organization 5-item Well-Being Index: WHO-5 [28]	
	Short depression screening tool of the WHO.
	Domain: Psychological well-being (mood, interests, energy, sleep, psychomotor functioning); time frame: Past 14 days.
	5 items, 6-point Likert scale from 0 to 5; total sum score from 0 to 25; higher scores indicating better well-being.
	Cut off score: WHO-5 sum score <14, clinically relevant depressive state [28].
Alcohol Use Disorder Identification Test:AUDIT [29], [30]	
	WHO screening tool for alcohol-related problems.
	Domain: Hazardous and harmful alcohol consumption, and alcohol-related problems; time frame: Past 12 months.
	10 items, 5-point Likert scale from 0 to 4; total sum score from 0 to 40. Applied scores in this study: AUDIT sum score for any alcohol use disorder, AUDIT-C score for risky alcohol consumption (sum of items 1 to 3).
	Cut off score: AUDIT sum score: ≥8 for men, ≥5 for women [29]; AUDIT-C score: >4 for men, >3 for women [30].

The evaluation of patients’ perioperative risk according to the ASA (American Society of Anesthesiologists) physical status classification system was used as an overall indicator for physical health [32], [33]. The evaluation was performed by the anesthesiologists who did the preoperative assessment. The ASA system classifies patients in one of four categories: (1) Healthy patient; (2) mild systemic disease, no functional limitation; (3) severe systemic disease with definite functional limitation; (4) severe systemic disease that is a constant threat to life. The fifths category which comprises moribund patients was not used in this study. Information on the surgical field was obtained from the electronic patient management system of the Charité – University Medicine Berlin and comprised the categories 1) abdomino-thoracic surgery, 2) peripheral surgery, 3) neuro, head and neck surgery. Data on ASA classification and surgical field were available for 715 patients in the implementation phase.

### Sample

Univariate analyses showed that the 1,157 participants differed from the 3,411 patients who did not participate in the follow-up with regard to interest in psychotherapy, demographic characteristics and diverse clinical factors (Table 2). A multivariate logistic regression model with the dependent variable ‘participant vs. nonparticipant’ and all variables with a significant effect in univariate analyses as covariates revealed that only four covariates continued to indicate significant differences (in order to prevent artificial effects, only the total AUDIT score but not the AUDIT-C subscore was included): Age *[OR 1.03 (95% CI 1.02–1.04), p<0.001]*, gender *[OR 1.43 (95% CI 1.18–1.74), p<0.001]*, interest in psychotherapy *[OR 1.65 (95% CI 1.23–2.20), p = 0.001]*, and university entrance qualification *[OR 1.26 (95% CI 1.05–1.51), p = 0.015]*.

**Table 2:** Comparisons of participants (n = 1,157) and nonparticipants (n = 3,411) of the 6-month follow-up; n (%); mean [SD].

	Participants n = 1,157+	Nonparticipants n = 3,411+	*p*
**Interest in psychotherapy**	193 (16.7)	336 (9.9)	<0.001
**Age: Years**	52.49 [15.53]	45.75 [16.02]	<0.001
**Women**	667 (57.6)	1708 (50.1)	<0.001
**Partnership status: Living with a partner**	768 (67.1)	2051 (60.8)	<0.001
**Level of education: University entrance qualification**	511 (44.6)	1374 (40.5)	0.015
**ASA Classification^++a)^**			<0.001
ASA I	172 (24.1)	773 (34.1)	
ASA II	416 (58.2)	1218 (53.8)	
ASA III	125 (17.5)	270 (11.9)	
ASA IV	2 (0.3)	5 (0.2)	
**Surgical field** ++			0.028
Abdomino thoracic surgery	283 (39.6)	868 (38.3)	
Peripheral surgery	235 (32.9)	660 (29.1)	
Neuro-, head and neck surgery	197 (27.6)	738 (32.6)	
**BSI GSI, severity of psychological distress** b)	0.34 [0.37]	0.33 [0.39]	0.168
**Self-rating of current subjective health** c)	64.31 [24.78]	64.22 [27.49]	0.917
**WHO-5** d)	14.54 [5.83]	14.44 [5.79]	0.587
**PHQ-2** e)	1.48 [1.44]	1.40 [1.40]	0.094
**CES-D** f)	9.98 [6.90]	9.56 [6.56]	0.078
**BSI depression** b)	0.32 [0.53]	0.30 [0.52]	0.377
**GAD-2** g)	1.35 [1.51]	1.23 [1.41]	0.013
**BSI anxiety** b)	0.39 [0.47]	0.35 [0.47]	0.008
**BSI interpersonal sensitivity** b)	0.35 [0.53]	0.34 [0.53]	0.347
**BSI phobic anxiety** b)	0.18 [0.38]	0.17 [0.37]	0.544
**AUDIT sum score** h)	2.79 [3.15]	3.09 [3.52]	0.007
**AUDIT-C: Risky alcohol consumption** h)	2.34 [1.96]	2.50 [2.13]	0.015

+ Number ranges for the specific variables from 1,123 to 1,157 (participants) and from 3,236 to 3,411 (nonparticipants) because of missing data.

++ Data for ASA and surgical field are available for the implementation phase; numbers account to 715 (participants), and 2266 (nonparticipants) because of missing data.

a) ASA (American Society of Anesthesiologists) physical status classification: (I) Healthy patient; (II) Mild systemic disease, no functional limitation; (III) Severe systemic disease with definite functional limitation; (IV) Severe systemic disease that is a constant threat to life;

b) BSI: Brief Symptom Inventory; GSI: General severity index;

c) Visual analogue scale of the EQ-5D, 0 to 100 with higher scores indicating better subjective health;

d) WHO-5: World Health Organization 5-item Well-Being Index;

e) PHQ-2: Patient Health Questionnaire-4, depression subscale;

f) CES-D: Center for Epidemiologic Studies Depression Scale;

g) GAD-2: Patient Health Questionnaire-4, anxiety subscale;

h) AUDIT: Alcohol Use Disorder Identification Test, AUDIT-C: AUDIT subscore for risky alcohol consumption.

### Statistical Analyses

Data were entered into a computerized database and statistical analyses were performed with SPSS Statistics for Windows, Version 19 (SPSS Inc., Chicago, Illinois 60606, USA). Results were expressed as relative frequencies in percent, mean (M) and standard deviation (SD), estimated marginal mean (EMM) and standard error of the mean (SEM), respectively. Participants and nonparticipants of the follow-up were first compared with univariate analyses by T test and Chi-squared test; in a second step, multivariate analyses were performed by entering all variables showing group differences with a p<.05 in univariate analyses into a logistic regression model; odds ratios [OR] with 95%-confidence intervals were given. A two-tailed p-value <0.05 was considered statistically significant. Comparison of patients with and without interest in psychotherapy were carried out with Chi-squared test for categorical and T test for metric data.

Change of psychological distress between baseline assessment (T1) and 6-month follow-up (T2) in patients with and without interest in psychotherapy was tested with McNemar’s test for categorical data and repeated measures ANCOVA for metric data. Repeated measures ANCOVA included as dependent variables the T1 and T2 measures of psychological distress, as within-subject factor the time points T1 and T2, as between-subject factors interest in psychotherapy, gender and partnership status, as well as age as covariate. Analyses were carried out twofold, on raw data of the questionnaires (ANCOVA), as well as regarding rates of cases with clinically significant psychological distress (McNemar’s test). A case with clinically significant distress was defined as a patient scoring above the cut off score of a given questionnaire; cut off scores of all measures are shown in Table 1. Bonferroni corrections were used in order to prevent the increase of type I error in ANCOVAs and McNemar’s tests. Analyzing 12 measures of psychological distress as dependent variables (BSI GSI, subjective health, WHO-5, PHQ-2, CES-D, BSI depression, GAD-2, BSI anxiety, BSI interpersonal sensitivity, BSI phobic anxiety, AUDIT sum score, AUDIT-C), a two-tailed p-value <0.0041 (0.05/12) was considered statistically significant for each of the 12 single tests of the ANCOVA model. Because there are no cut off scores indicating clinically significant distress of the variable ‘subjective health’, only 11 of the 12 measures of psychological distress were analyzed as dependent variables of the McNemar’s tests. As a consequence, a two-tailed p-value <0.0045 (0.05/11) was considered statistically significant for each of the 11 single McNemar’s tests.

## Results

Out of all 1,157 participants, 193 patients (16.7%) were interested in psychotherapy, and 964 (83.3%) were not interested. Patients with interest in psychotherapy were statistically significantly younger (p<0.001), were more likely to be female (p = 0.012) and less likely to live with a partner (p<0.001). However, there was no significant difference regarding surgical field (p = 0.731) and ASA classification (p = 0.122) (Table 3).

**Table 3:** Sociodemographic and clinical characteristics of all participants of the 6-month follow-up (N = 1,157), as well as comparison of patients who showed interest in psychotherapy (n = 193) and patients who were not interested in psychotherapy (n = 964); mean [SD], n (%).

	All participantsN = 1,157+	Patients interestedin psychotherapyn = 193+	Patients notinterested inpsychotherapyn = 964+	*p*
**Sociodemographic characteristics**				
Age: Years	52.49 [15.53]	48.95 [13.61]	53.19 [15.80]	<0.001
Male	490 (42.40)	66 (34.20)	424 (44.00)	0.012
Partnership status: Living with a partner	768 (67.10)	106 (56.10)	662 (69.20)	<0.001
Level of education: University entrance qualification	511 (44.60)	93 (48.40)	418 (43.90)	0.245
**Clinical characteristics**				
ASA Classification^++a)^				0.122
ASA I	172 (24.10)	18 (17.80)	154 (25.10)	
ASA II	416 (58.20)	58 (57.40)	358 (58.30)	
ASA III	125 (17.50)	25 (24.80)	100 (16.30)	
ASA IV	2 (0.30)	0 (0.00)	2 (0.30)	
Surgical field++				0.731
Abdomino thoracic surgery	283 (39.60)	43 (42.60)	240 (39.10)	
Peripheral surgery	235 (32.90)	30 (29.70)	205 (33.40)	
Neuro-, head and neck surgery	197 (27.60)	28 (27.70)	169 (27.50)	

+ Number ranges for the specific variables from 1,145 to 1,157 (all participants), from 189 to 193 (patients interested in psychotherapy) and from 953 to 964 (patients not interested in psychotherapy) because of missing data.

++ Data for ASA and surgical field are available for the implementation phase; numbers account to 715 (all participants), 101 (patients interested in psychotherapy, and 614 (patients not interested in psychotherapy) because of missing data.

a) ASA (American Society of Anesthesiologists) physical status classification: (I) Healthy patient; (II) Mild systemic disease, no functional limitation; (III) Severe systemic disease with definite functional limitation; (IV) Severe systemic disease that is a constant threat to life.

In order to control for the differences in sociodemographic characteristics, the ANCOVAs analyzing the course of self-reported psychological distress include gender and partnership status as additional between-subject factors and age as a covariate; results are shown in Table 4. Patients with interest in psychotherapy showed considerably higher distress than patients without interest in psychotherapy at baseline and at month 6 regarding all 12 measures of distress, with p’s between <0.001 and 0.003 for the different comparisons (Table 4). Importantly, neither of the two groups showed statistically significant changes over time in 11 scales. Only in one scale a small but statistically significant change occurred: Both groups had a slight increase in BSI interpersonal sensitivity (p<0.001). Significant interaction effects were only observed for GAD-2 (p = 0.002) and BSI anxiety (p = 0.002) with a stronger decrease in both anxiety scales in patients with interest in psychotherapy.

**Table 4:** 6-month follow-up of psychological distress and alcohol use problems of surgical patients with interest in psychotherapy (n = 193) and surgical patients without interest in psychotherapy (n = 964).+

	Patients with interest in therapy contacts n = 193++		Patients without interest in therapy contacts n = 964++				
	T1	T2	T1	T2	*Group*	*Time*	*Group x Time*
	*EMM (SEM)*	*EMM (SEM)*	*EMM (SEM)*	*EMM (SEM)*	*P*	*P*	*P*
**General psychological distress and subjective health**							
BSI GSI, severity of psychological distressa)	0.70 (0.03)	0.72 (0.03)	0.28 (0.01)	0.33 (0.02)	<0.001	0.005	0.307
Self-rating of current subjective healthb)	54.18 (1.95)	56.43 (1.85)	65.95 (0.91)	69.13 (0.86)	<0.001	0.250	0.707
**Depression**							
WHO-5c)	10.97 (0.43)	11.48 (0.45)	15.22 (0.20)	15.40 (0.21)	<0.001	0.603	0.511
PHQ-2d)	2.55 (0.11)	2.33 (0.11)	1.29 (0.05)	1.35 (0.05)	<0.001	0.111	0.025
CES-De)	15.51 (0.50)	15.61 (0.63)	8.98 (0.23)	8.64 (0.30)	<0.001	0.776	0.479
BSI depression a)	0.79 (0.04)	0.82 (0.05)	0.25 (0.02)	0.33 (0.02)	<0.001	0.0043	0.423
**Anxiety**							
GAD-2f)	2.51 (0.11)	2.06 (0.11)	1.10 (0.05)	1.03 (0.05)	<0.001	0.704	0.002
BSI anxietya)	0.77 (0.03)	0.62 (0.03)	0.31 (0.02)	0.28 (0.02)	<0.001	0.622	0.002
BSI interpersonal sensitivitya)	0.77 (0.04)	0.82 (0.04)	0.28 (0.02)	0.32 (0.02)	<0.001	<0.001	0.846
BSI phobic anxietya)	0.45 (0.03)	0.43 (0.04)	0.12 (0.01)	0.15 (0.02)	<0.001	0.037	0.164
**Alcohol problems**							
AUDIT sum score: Alcohol abuse/dependenceg)	4.08 (0.24)	4.15 (0.25)	2.78 (0.11)	2.86 (0.12)	<0.001	0.217	0.952
AUDIT-C: Risky alcohol consumptiong)	2.90 (0.15)	2.70 (0.15)	2.35 (0.07)	2.35 (0.07)	0.003	0.675	0.077

+ Repeated measures ANCOVA; estimated marginal means (EMM) and standard error of the mean (SEM); statistical significance of Bonferroni correction: p<0.0041; T1 (preoperative baseline assessment) and T2 (postoperative 6 months follow-up) as within-subject factors, therapy interest as between subject factor; covariate: age; additional between-subject factors: gender and partnership status.

++ Number ranges for the specific variables from 173 to 187 (patients interested in therapy contacts), and from 878 to 938 (patients not interested in therapy contacts) because of missing data

a) BSI: Brief Symptom Inventory; GSI: General severity index

b) Visual analogue scale of the EQ-5D, 0 to 100 with higher scores indicating better subjective health

c) WHO-5: World Health Organization 5-item Well-Being Index

d) PHQ-2: Patient Health Questionnaire-4, depression subscale

e) CES-D: Center for Epidemiologic Studies Depression Scale

f) GAD-2: Patient Health Questionnaire-4, anxiety subscale

g) AUDIT: Alcohol Use Disorder Identification Test, AUDIT-C: AUDIT subscore for score for risky alcohol consumption

Similar results were found with regard to clinically relevant psychological symptoms (details in Table 5). In patients with interest in psychotherapy, rates of clinically significant symptoms were high and stable between baseline and month 6 regarding all tested measures. Patients without interest in psychotherapy showed low rates of clinically significant distress that also remained stable between baseline and month 6. Interestingly, rates even increased statistically significantly in two scales, BSI GSI (p = 0.001) and BSI depression (p = 0.001).

**Table 5:** 6-month follow-up of rates of clinically significant distress and alcohol use problems of surgical patients with interest in psychotherapy (n = 193) and surgical patients without interest in psychotherapy (n = 964).+

	Patients with interest in therapy contacts n = 194++			Patients without interest in therapy contacts n = 966++		
	T1	T2	*Time*	T1	T2	*Time*
	n (%)	n (%)	*P*	n (%)	n (%)	*P*
**General psychological distress**						
BSI GSI, severity of psychological distressa)	88 (46.8)	83 (44.1)	.576	98 (10.5)	135 (14.4)	.001
**Depression**						
WHO-5b)	123 (64.4)	111 (58.1)	.162	302 (32.1)	291 (30.9)	.505
PHQ-2c)	82 (43.4)	64 (33.9)	.041	131 (14.1)	141 (15.2)	.490
CES-Dd)	71 (38.4)	65 (35.1)	.497	86 (9.4)	116 (12.6)	.005
BSI depression a)	73 (38.2)	72 (37.7)	1.00	88 (9.4)	121 (12.9)	.001
**Anxiety**						
GAD-2e)	71 (38.0)	52 (27.8)	.018	106 (11.6)	80 (8.8)	.018
BSI anxiety a)	71 (37.4)	49 (25.8)	.005	83 (8.8)	75 (8.0)	.475
BSI interpersonal sensitivitya)	53 (27.7)	56 (29.3)	.761	54 (5.8)	64 (6.8)	.268
BSI phobic anxietya)	58 (30.7)	47 (24.9)	.108	69 (7.3)	88 (9.3)	.040
**Alcohol problems**						
AUDIT sum score: Alcohol abuse/dependencef)	35 (18.6)	35 (18.6)	1.00	83 (8.9)	86 (9.3)	.810
AUDIT-C: Risky alcohol consumptionf)	49 (26.3)	43 (23.1)	.362	164 (18.1)	148 (16.3)	.137

+ McNemar’s test; T1: Preoperative baseline assessment; T2: Postoperative 6 months follow-up; n (%); statistical significance of Bonferroni correction: p<0.0045.

A case with clinically significant distress was defined as a patient scoring above the cut off score of a given questionnaire (see Table 1 for cut off scores of all measures).

++ Number ranges for the specific variables from 185 to 191 (patients interested in therapy contacts), and from 907 to 943 (patients not interested in therapy contacts) because of missing data.

a) BSI: Brief Symptom Inventory; GSI: General severity index;

b) WHO-5: World Health Organization 5-item Well-Being Index;

c) PHQ-2: Patient Health Questionnaire-4, depression subscale;

d) CES-D: Center for Epidemiologic Studies Depression Scale;

e) GAD-2: Patient Health Questionnaire-4, anxiety subscale;

f) AUDIT: Alcohol Use Disorder Identification Test, AUDIT-C: AUDIT subscore for score for risky alcohol consumption.

## Discussion

To the authors’ knowledge, this is the first long-term study on psychological distress in surgical patients that included interest in psychotherapy as a group factor. The results revealed that (1) patients with interest in psychotherapy differed considerably from patients without interest in psychotherapy; (2) there were no substantial changes of distress between preoperative assessment and 6-month follow-up in both patient groups. The most important finding is that interested patients showed consistently high distress at both baseline and month 6 regarding all of the assessed psychological measures of general distress, depression, anxiety, subjective health, and alcohol use disorder symptoms. This remarkable persistence suggests high and chronic psychiatric comorbidity and a clear need for psychotherapy rather than transient worries posed by facing surgery. These results might be considered as unsurprising in a setting of psychosocial health care. However, data were collected in preoperative anesthesiological assessment clinics where surgical patients are examined by an anesthesiologist to clarify anesthesia related risks of the intended surgery and to evaluate the patients’ individual level of risk. In this setting, patients prepare to undergo surgery and both patients and clinicians do not expect psychological screening programs. Clinically significant preoperative psychological distress may be misinterpreted by anesthesiologists and surgeons as transient worries about somatic diagnoses and the forthcoming surgery. Thus, it is important to provide evidence that patients with high preoperative psychological distress and interest in psychotherapy do not easily improve after having overcome surgery and the hospital stay. From a psychotherapeutic perspective it makes sense to treat chronic psychiatric comorbidity in surgical patients who are motivated for therapy. But also from a medical perspective this implication becomes comprehensive: Recent studies provided evidence that untreated depression, anxiety and substance use disorders are associated with perioperative complications and increased morbidity and mortality, leading to worse surgical outcomes and higher health care costs of surgical patients [3]–[9], [34]–[38]. In order to properly assess and treat psychological distress in surgical patients, cost-efficient approaches are needed that are based on interdisciplinary collaboration of clinicians from anesthesiology, surgery and psychology. A stepped care program may fulfil both clinical and economical demands of such an approach [23]: Screening for psychological distress, brief motivational interventions, as well as early supportive interventions for transiently elevated perioperative distress can be performed by psychologically trained nursing staff. After the screening, those patients who wish to be visited by a psychotherapist may communicate their interest to the nursing staff to arrange a first appointment. Non-confrontational brief advice should be offered to patients who show clinically significant distress but lack motivation for therapy. The data of the present study suggest that patients with both clinically significant preoperative psychological distress and the explicit interest in psychotherapy are at an increased risk to have persistently high distress after 6 months. As a consequence, for these patients, the therapeutic steps after the screening should comprise detailed psychological assessment, clarification of psychiatric diagnoses according to ICD-10, first psychotherapy sessions including motivational interviewing, and, if required, the initiation of longer psychosocial treatment options.

The lack of any substantial changes of psychological distress over time that was found in this study is in agreement with previous studies in small samples of specific surgical patient groups that found no significant change of clinically relevant depression and anxiety during follow-up times between 1 month and 12 months, e.g. [12]–[14]. The slight decrease of anxiety in patients with interest in psychotherapy can be considered as being consistent with studies that reported a decrease of state anxiety between 3 days and 3 months postoperatively without examining clinically significant anxiety [16], [20]. However, there is a clear contradiction to investigations that observed a clear and significant postoperative decrease of clinically significant depression and anxiety during 6 months, e.g. [15], [17], [18]. In patients who were not interested in psychotherapy, there seems to be an increase of some clinically significant symptoms of general distress and depression by month 6. This partial result is consistent with the findings of O’Hara et al (1989) [1], as well as Gallagher & McKinley (2009) [10] and Tsapakis et al (1989) [11] who found a significant increase of psychiatric symptomatology in the postoperative period with follow-up times between 8 and 12 weeks. Finally, the results are partly inconsistent with the findings of Krannich et al (2007) [19] who observed no significant changes regarding the rates of clinically relevant depression and anxiety but a significant decrease when analyzing raw data of the Hospital Anxiety and Depression Scale (HADS) with T tests.

### Methodological Limitations

This study is a substudy of the feasibility study of BRIA that did not strictly focus on long-term investigation. As a consequence, a large number of patients failed to participate in the follow-up so that an effect of self-selection has to be assumed for this substudy. Univariate comparison of participants and nonparticipants of the follow-up showed that participants were significantly older, were more likely to be female, to live with a partner, to be with university entrance qualification, and that they had worse preoperative physical health. Additionally, they scored higher regarding general anxiety but they reported less alcohol use problems. Importantly, the percentage of patients with interest in psychotherapy was higher in participants (16.7%) than in nonparticipants (9.9%). On the other hand, there were no significant differences regarding subjective health and most domains of psychological distress, depression and anxiety. Multivariate analyses identified only four important factors that characterized study participants: Higher age, as well as higher percentages of women and patients with interest in psychotherapy and with university entrance qualification. About the reasons why participants and nonparticipants differed regarding these characteristics can only be speculated. A self-selection of patients with more severe psychiatric symptoms can be ruled out because both groups did not differ substantially regarding preoperative psychological distress. On the other hand, it might be assumed that the characteristics associated with participation are related to diverse personality factors, like conscientiousness, interest in psychological research, or the need to disclose ones status of subjective health, patient satisfaction and quality of life. To conclude, generalization is a major methodological limitation of this study. It is open whether the results can be generalized to samples with participants who are younger and who are more likely to be men, without university entrance qualification and who are less likely to show interest in psychotherapy.

Another issue that has to be mentioned is the use of self-report data. Results based on standardized and validated questionnaires can be assumed to correctly indicate chronic psychiatric comorbidity. However, questionnaire measures do not represent diagnoses of specific mental disorders. Only detailed psychological assessment and structured clinical interviews will clarify whether patients scoring above a given questionnaire cut off have diagnoses according to ICD-10 or DSM-IV-R. Even though studies on sensitivity and specificity of the applied screening tools exist for the general medical field, they are still missing for the perioperative setting.

### Clinical Implications

There are four major clinical implications of this study. (1) The finding of stable and chronic psychiatric comorbidity and its association with interest in psychotherapy suggests an implementation of the assessment of psychological problems and their treatment in routine care of anesthesiology and surgery. (2) There is no evidence that elevated preoperative symptoms of psychological distress in surgical patients are only signs of transient worries that are easily overcome by spontaneous remission. Depression, anxiety and substance use disorders should be considered as serious comorbid conditions in these patients that deserve adequate treatment. (3) Because the wish for psychotherapy seems to be independent from surgical field and preoperative physical health, clinicians should be encouraged not to restrict screening for need of therapy and psychological distress to patients from specific surgical fields or with specific medical diagnoses. (4) The chronicity of psychological distress suggests the application of comprehensive empirically supported psychotherapy. Screening and successful motivational interventions are the first steps to help patients to engage and maintain psychotherapy programs that aim at recovery from depression, anxiety and substance use disorders.
